# Comparing fine motor performance among young children with autism spectrum disorder, intellectual disability, attention-deficit/hyperactivity disorder, and specific developmental disorder of motor function

**DOI:** 10.3389/fped.2024.1372980

**Published:** 2024-03-18

**Authors:** Ling-Yi Lin, I-Ting Hwang, Chia-Fen Hsu, Wen-Hao Yu, Pei-Chun Lai, Yi-Wen Chen, Yi-Fang Tu

**Affiliations:** ^1^ Department of Occupational Therapy, College of Medicine, National Cheng Kung University, Tainan, Taiwan; ^2^ Institute of Allied Health Sciences, College of Medicine, National Cheng Kung University, Tainan, Taiwan; ^3^ Department of Pediatrics, National Cheng Kung University Hospital, College of Medicine, National Cheng Kung University, Tainan, Taiwan; ^4^ Institute of Clinical Medicine, College of Medicine, National Cheng Kung University, Tainan, Taiwan; ^5^ Educational Center, National Cheng Kung University Hospital, College of Medicine, National Cheng Kung University, Tainan, Taiwan; ^6^ Department of Nursing, National Cheng Kung University Hospital, College of Medicine, National Cheng Kung University, Tainan, Taiwan

**Keywords:** fine motor, autism spectrum disorder, intellectual disability, attention-deficit/hyperactivity disorder, developmental delay

## Abstract

**Objective:**

The acquisition of fine motor skills is considered to be a crucial developmental milestone throughout early childhood. This study aimed to investigate the fine motor performance of young children with different disability diagnoses.

**Methods:**

We enrolled a sample of 1,897 young children under the age of 6 years who were at risk of developmental delays and were identified by a transdisciplinary team. A series of standardized developmental assessments included the Bayley Scales of Infant Development-Third Edition, Wechsler Preschool and Primary Scale of Intelligence-Fourth Edition, Peabody Developmental Motor Scale-Second Edition, and Movement Assessment Battery for Children-Second Edition were used. Retrospective chart reviews were conducted on all children to identify specific developmental disorders. The number of autism spectrum disorder (ASD), intellectual disability (ID), attention-deficit/hyperactivity disorder (ADHD), comorbidity, motor dysfunction, and unspecified developmental delays (DD) were 363 (19.1%), 223 (11.8%), 234 (12.3%), 285 (15.0%), 128 (6.7%), and 590 (31.1%), respectively.

**Results:**

Young children with ID, comorbidity, and motor dysfunction demonstrated significant difficulty in performing manual dexterity and visual motor integration tasks and scored significantly lower in these areas than children with ASD, ADHD, and unspecified DD. In addition, fine motor performance was associated with cognitive ability in children with different disability diagnoses, indicating that young children showed better fine motor performance when they demonstrated better cognitive ability.

**Conclusion:**

Our findings support that differences in fine motor performance differ by disability type. Close links between fine motor performance and cognitive ability in children under the age of 6 years were seen in all disability types.

## 1 Introduction

Young children experience rapid growth in various domains, including motor, language, cognitive, social, and emotional development during early childhood (1). Fine motor skills are critical for many daily activities, such as manipulating toys or objects, dressing, and grooming. Currently, the percentage of children using touchscreen devices (e.g., smartphones and tablets) has been increasing and many children acquire additional actions: tapping, double-tapping, pressing, sweeping, dragging, and zooming when using touchscreen devices (2). However, the frequent use of touchscreen devices may limit the fine motor development of typically developing young children (3). Little evidence suggests that using a touchscreen device is related to the fine developmental skills of young children with developmental disabilities. It is unclear whether children with developmental disabilities frequently use touchscreen devices. In addition, several factors are associated with the fine motor development of children. In the past 20 years, researchers have increasingly focused on the fine motor skills of children with developmental disabilities and how these skills relate to their cognitive ability (4–6). Although some research exists on the correlation between fine motor development and cognitive ability in Western countries, there is a lack of such studies in Asian countries.

In the United States, the most common developmental disabilities include attention-deficit/hyperactivity disorder (ADHD), intellectual disability (ID), autism spectrum disorder (ASD), and other developmental delay (DD) with a prevalence of 9.57% in ADHD, 2.94% in ASD, 1.72% in ID, and 5.24% in other DD from 2018 to 2021 (7). It has been found that developmental disabilities such as ID, ASD, and ADHD often co-occur (7). Recent studies show that children with developmental disabilities have below-average fine motor skills compared to their age group (4, 5). Children with developmental disabilities also tend to have more motor impairments than typically developing (TD) children (4, 5). For instance, children with ASD perform fine motor skills worse than TD children (8–10). Similar findings have been reported in children with ID or ADHD (11–13). However, it is important to note that individual differences in specific symptoms and concurrent comorbidity are common among individuals with developmental disabilities and should not be overlooked.

Moreover, research on motor differences of children with ASD compared with other children with developmental concerns has been inconclusive. When comparing children with different types of diagnosis, children with ASD performed more poorly than children with ADHD (13) or DD (14), whereas others observed no such difference (4, 15, 16). According to Jeoung (17), there were no notable variations in fine motor skills between children with ASD and those with borderline or mild ID. However, children with moderate ID scored significantly lower than children with ASD in fine motor precision, fine motor integration, and manual dexterity. Thus far, it is not known whether young children with different types of developmental disabilities differ in their fine motor development in comparison to TD children and children with developmental concerns. Furthermore, most prior research on fine motor skills has been conducted with sample sizes of less than 212 participants (ranging from 38 to 212). Those participants were across a wide age range from 12 months to 16 years, primarily for school-aged children with developmental disabilities. To date, few studies have mainly focused on the comparison of fine motor performance below the age of 6 years.

One of the commonly discussed factors related to fine motor performance is cognitive ability. A relationship between motor and cognitive development among children is evident and consistent with this perspective (18, 19). It has been observed that children with poor fine motor skills often exhibit poor cognitive ability and those with cognitive delays are more likely to experience motor difficulties (18, 19). Fine motor skills facilitate visuospatial cognition and exploratory behaviors (20). Previous studies indicated that fine motor skills are associated with cognitive ability in children with ID (6, 21), ASD (22, 23), and ADHD (24). However, the different patterns of associations between fine motor skills and cognitive skills for children with ASD co-occurring ID or ADHD and specific developmental disorders of motor function were not clarified. For instance, manual dexterity was not associated with planning and attention cognitive skills for children with developmental coordination disorder (25). There were inconclusive results regarding the relationship between fine motor skills and cognitive ability and this may need further investigation.

Various individual characteristics have been associated with fine motor development, and previous studies have shown conflicting results regarding the influence of birth order on motor development during childhood (26, 27). For example, Krombholz (26) reported that first-born children exhibit better fine motor development than later-born children. On the other hand, Rebelo et al. (27) found that later-born children tend to have higher fine motor performance compared to first-born children. As there are no other studies available to date that can confirm the impact of birth order on fine motor development, there is insufficient information to determine the role of birth order in the fine motor development of young children with different disability diagnoses.

Existing studies show limitations, such as small sample sizes, wide age ranges, and inclusion of children with one specific developmental concern and heterogeneous characteristics. Therefore, it is necessary to improve our understanding of fine motor performance and its relation to cognitive ability in children with different developmental concerns. This study aimed to compare the fine motor performance between children with different disability diagnoses and TD children. In this study, we address the following questions: (a) Do differences exist in the fine motor performance between children with different disability diagnoses and TD children? (b) Do differences exist in the fine motor performance among young children with different disability diagnoses? (c) What relationships exist between fine motor performance and cognitive ability among young children with different disability diagnoses? It may be useful for clinicians to understand how fine motor skills relate to cognitive ability, as this can help design targeted interventions for different disabilities.

## 2 Methods

### 2.1 Design

A retrospective cohort study was conducted.

### 2.2 Participants

Data were drawn from the Center of Team Evaluation for Children's Development in southern Taiwan from January 2018 to December 2022. The Center of Team Evaluation for Children's Development served children under 6 years at risk for/with developmental delay. In Taiwan, if parents had concerns about the condition of their child due to developmental disorders and health, they could ask pediatricians for a referral to the center for a comprehensive evaluation. Some children were automatically referred by pediatricians while at the clinic visit or by teachers when they failed the annual developmental screening in preschools. All children who were at risk of developmental delays would be referred to the center for a comprehensive evaluation by a transdisciplinary team. A transdisciplinary team comprising a pediatric neurologist, a pediatric psychiatrist, three psychologists, two occupational therapists, two physical therapists, and one speech therapist conducted a series of standardized developmental assessments for all children. After the comprehensive developmental evaluation by a transdisciplinary team, the pediatric neurologist and pediatric psychiatrist would give the diagnosis to children based on the ICD-10 codes and the Diagnostic and Statistical Manual of Mental Disorders-Fifth Edition. The diagnoses were documented on the medical chart. The dataset consists of 2,813 children. Children with previous diagnoses of diseases or disorders (e.g., cerebral palsy, chromosomal anomalies or abnormalities, hypoxic–ischemic encephalopathy, traumatic brain injury, meningitis; *N *= 617) were excluded from the analysis. Additionally, children who did not have fine motor assessment data were excluded (*N* = 299). Finally, a sample of 1,897 children at risk for/with developmental delay were enrolled in this study. Two different developmental assessments were used for two separate age groups. The participants were divided into two groups based on their age bands. Out of the 1,897 children, 1,424 were aged 36 months and above, and 473 were under the age of 3 years.

#### Group 1

A sample of 1,424 children aged between 36 and 77 months (*M* = 52.5, SD = 9.5) were recruited, of whom 1,035 (72.7%) were boys. Their cognitive ability ranged from 40 to 130 on the Wechsler Preschool and Primary Scale of Intelligence, with a mean score of 84.4, and 15.3% of them scored below 70.

#### Group 2

A total of 473 children under the age of 3 years (8–35 months) were recruited, with a mean age of 26.9 months. Almost two-thirds of the sample were boys (66.3%). Their cognitive ability ranged from 53 to 140 on the Bayley Scales of Infant Development-Third Edition (Bayley-III), with a mean score of 84.7, and 39.5% of them scored below 85.

### 2.3 Measures

A series of developmental assessments were administered to all children in a standardized manner. A transdisciplinary team used instruments including the Wechsler Preschool and Primary Scale of Intelligence-Fourth Edition (WPPSI-IV), Bayley Scales of Infant Development-Third Edition (Bayley-III), Movement Assessment Battery for Children-Second Edition (MABC-2), and Peabody Developmental Motor Scale-Second Edition (PDMS-2). These instruments were used to identify the cognitive and motor abilities of children. The WPPSI-IV (28) and Bayley-III (29) demonstrated good to excellent reliability and validity. The registered psychologists administered the Bayley-III and WPPSI-R. Occupational therapists and physical therapists tested the PDMS-2 and MABC-2. The investigator conducted retrospective chart reviews on all children to confirm a diagnosis of ASD (F84.0 and F84.9), ID (F70, F71, and F72), ADHD (F90.0 and F90.2), and motor dysfunction (F82) based on the ICD-10 codes. More than one specific diagnosis (e.g., ASD comorbid ID or ADHD) was categorized as comorbidity. The rest of the participants at risk for/with developmental delay were identified as unspecified developmental delay (DD).

The PDMS-2 (30) was used to assess the fine motor (FM) skills and ability of children younger than 36 months old, including grasping and visual–motor integration subtests. Standard and quotient scores of the FM subtest were analyzed. Children with a quotient below 85 were described as developmentally delayed. In terms of two subtests, at risk for/with motor delays was defined as a standard score below 7. The PDMS-2 has shown high test–retest reliability and acceptable responsiveness among Taiwanese children (31). The MABC-2 was used to assess fine motor skills for children at or above the age of 3 years. The manual dexterity subtests were administered by occupational therapists. Standard scores of the subtest were analyzed. At risk for/with motor difficulties was defined as a standard score below 7. The MABC-2 has reported good reliability and validity (32).

### 2.4 Procedures

The study protocol was approved by the Institutional Review Board (A-ER-111-067) at National Cheng Kung University Hospital. Initially, the case manager obtained written informed consent and interviewed the caregivers for their concerns and demographic characteristics (e.g., age, gender, birth order, medical history, parental education level). The caregivers were also asked whether or not the child used the smartphone (1 = yes, 0 = no). Next, the WPPSI-IV or Bayley-III was administered by a qualified psychologist. The MABC-2 or PDMS-2 was administered by an occupational therapist. Then, the pediatric neurologist and pediatric psychiatrist finalized the diagnosis of the children.

### 2.5 Data analysis

Descriptive statistics (such as frequencies, percentiles, means, and standard deviations) were analyzed using SPSS 25.0. The chi-squared test and one-factor analysis of variance were examined, with the significance level set at 0.05. The chi-squared tests were used to determine the differences in the categorical variables, and the ANOVA tests followed by Bonferroni's *post hoc* comparison tests were used to examine the group differences in other variables. We also used MANOVA tests followed by Bonferroni's *post hoc* comparisons to compare the groups on the MABC-2 and PDMS-2 subtests. Pearson’s correlation coefficients were used to examine the relationships between cognitive ability and fine motor performance among these participants.

## 3 Results

Table 1 presents the demographic characteristics of the participants. The participants were divided into two groups based on the different developmental assessments used in two different age bands. For Group 1, the number of ASD (*N* = 275; 19.3%), ID (*N* = 124; 8.7%), ADHD (*N* = 234; 16.4%), comorbidity (*N* = 212; 14.9%), and motor dysfunction (*N* = 109; 7.7%) was identified in 1,424 children at risk for/with developmental delay. There were 52 children identified as typically developing (TD). Almost two-thirds of the children were the first child in the family, and 64.4% of the children used smartphones. For Group 2, the number of ASD (*N* = 88; 18.6%), ID (*N* = 99; 20.9%), and comorbidity (*N* = 73; 15.4%) was identified in 473 children at risk for/with developmental delay. There were 22 children identified as TD. Almost 63.8% of children were the first child in the family, and 43.3% used smartphones.

**Table 1:** Sample characteristics.

Variables	Group 1							
	ASD (*N* = 275)	ID (*N* = 124)	ADHD (*N* = 234)	Comorbidity (*N* = 212)	Motor dysfunction (*N* = 109)	Unspecified DD (*N* = 418)	TD (*N* = 52)	*χ*^2^ or F
Age (months)	52.0 (9.1)	50.0 (10.5)	55.2 (8.8)	53.3 (9.0)	53.1 (10.6)	51.7 (9.2)	52.0 (9.0)	6.04***
Age range	36–73	36–74	36–71	36–73	36–77	36–77	37–71	
Sex								
Male	228 (82.9%)	83 (66.9%)	173 (73.9%)	171 (80.7%)	68 (62.4%)	277 (66.3%)	35 (67.3%)	38.48***
Female	47 (17.1%)	41 (33.1%)	61 (26.1%)	41 (19.3%)	41 (37.6%)	141 (33.7%)	17 (32.7%)	
IQ (WPPSI-IV)	88.7 (12.4)	61.9 (7.9)	89.1 (11.9)	71.6 (16.6)	87.1 (11.7)	88.1 (11.8)	98.0 (9.2)	113.45***
IQ range	58–127	40–69	70–129	40–122	70–118	70–130	85–122	
Birth order-first	191 (69.5%)	78 (62.9%)	158 (67.5%)	136 (64.2%)	57 (52.3%)	233 (55.7%)	23 (44.2%)	28.46***
Using smartphone	157 (57.1%)	76 (61.3%)	148 (63.2%)	134 (63.2%)	69 (63.3%)	252 (60.3%)	35 (67.3%)	3.00
	Group 2							
	ASD (*N* = 88)	ID (*N* = 99)	Comorbidity (*N* = 73)	Motor dysfunction (*N* = 19)	Unspecified DD (*N* = 172)		TD (*N* = 22)	*χ*^2^ or F
Age (months)	28.5 (4.1)	24.5 (6.8)	27.4 (4.9)	23.9 (7.5)	27.6 (6.0)		26.0 (6.5)	11.50***
Age range	18–35	9–35	13–35	11–34	9–35		8–35	
Sex								
Male	68 (77.3%)	66 (66.7%)	58 (79.5%)	12 (63.2%)	109 (63.4%)		14 (63.6%)	11.30*
Female	20 (22.7%)	33 (33.3%)	15 (20.5%)	7 (36.8%)	63 (36.6%)		8 (36.4%)	
IQ (Bayley-III)	92.1 (6.8)	71.8 (8.6)	70.0 (7.6)	92.8 (6.6)	93.2 (9.0)		97.9 (6.9)	161.98***
IQ range	75–110	53–83	55–83	80–105	80–140		90–115	
Birth order	59 (67.0%)	59 (59.6%)	54 (74.0%)	13 (68.4%)	101 (58.7%)		16 (72.7%)	7.27
Using smartphone	45 (51.1%)	39 (39.4%)	29 (39.7%)	9 (47.4%)	75 (43.6%)		8 (36.4%)	7.85

WPPSI-IV, Wechsler Preschool and Primary Scale of Intelligence-Fourth Edition; Bayley-III, Bayley Scales of Infant and Toddler Development-Third Edition; IQ, intelligence quotient.

Group 1 age: ADHD = comorbidity = motor dysfunction **>TD = ASD = unspecified DD = ID.

Group 1 IQ: TD ***>ADHD = ASD = unspecified DD = motor dysfunction ***>comorbidity ***>ID.

Group 2 age: ASD = unspecified DD = comorbidity = TD ***>ID = motor dysfunction.

Group 2 IQ: TD ***>unspecified DD = motor dysfunction = ASD ***>ID = comorbidity.

* *p* < .05.

*** *p *< 0.001.

Table 2 summarizes the fine motor performance among these children. A MANOVA revealed that there was a significant main effect of group for the combined three subtests of the MABC-2 [Wilks’ lambda (*Λ*) = 0.741, *F* = 24.8, *p* < 0.001] and the combined two subtests of the PDMS-2 [Wilks’ lambda (*Λ*) = 0.618, *F* = 25.4, *p* < 0.001]. For both groups, TD children had significantly higher scores on all subtests of MABC-2 and PDMS-2 than those of the children with different disability diagnoses, except for children with unspecified DD. There was a significant main effect of the group on MABC-2 and PDMS-2 subtests, but not birth order or smartphone usage, and there was no interaction between groups and birth order or smartphone usage.

**Table 2:** Fine motor performance among children with different disability diagnoses.

Tests	Group 1							
	ASD (*N* = 275)	ID (*N* = 124)	ADHD (*N* = 234)	Comorbidity^a^ (*N* = 212)	Motor dysfunction (*N* = 109)	Unspecified DD (*N* = 418)	TD (*N* = 52)	F
MABC-2								
Placing coins	7.8 (2.7)	5.1 (3.1)	8.1 (3.1)	5.9 (3.2)	6.5 (3.1)	8.5 (2.9)	9.4 (2.1)	40.8***
Threading beads	7.7 (2.9)	5.0 (3.1)	8.1 (3.0)	5.5 (3.2)	6.2 (3.1)	8.4 (3.0)	10.1 (2.4)	48.1***
Drawing trail	7.3 (3.9)	3.3 (3.2)	7.8 (3.6)	4.6 (3.8)	5.3 (3.9)	8.0 (3.8)	8.8 (3.0)	48.2***
Manual dexterity	7.4 (2.7)	4.1 (2.4)	7.9 (2.9)	5.1 (2.7)	5.6 (2.7)	8.3 (3.1)	9.7 (1.9)	73.1***
	Group 2							
	ASD (*N* = 88)	ID (*N* = 99)	Comorbidity (*N* = 73)	Motor dysfunction (*N* = 19)	Unspecified DD (*N* = 172)	TD (*N* = 22)	F	
PDMS-2								
Grasping	8.6 (1.2)	7.4 (2.2)	8.1 (1.5)	6.8 (1.9)	8.8 (1.4)	9.0 (1.0)	15.2***	
VMI	7.5 (1.6)	5.7 (1.4)	5.3 (1.6)	5.6 (1.2)	8.4 (1.9)	9.6 (1.4)	65.2***	
FMQ	88.5 (6.1)	79.8 (8.9)	80.1 (7.0)	77.4 (5.0)	91.2 (9.7)	96.1 (5.7)	43.8***	

Placing coins: TD = unspecified DD*>ADHD = ASD**>motor dysfunction = comorbidity = ID.

Threading beads: TD **>unspecified DD = ADHD *>ASD ***>motor dysfunction = comorbidity = ID.

Drawing trail: TD = unspecified DD = ADHD = ASD ***>motor dysfunction = comorbidity = ID.

Manual dexterity: TD = unspecified DD **>ADHD = ASD ***>motor dysfunction = comorbidity = ID.

Grasping: TD = unspecified DD = ASD = comorbidity ***>ID = motor dysfunction.

VMI: TD = unspecified DD***>ASD***>ID = motor dysfunction = comorbidity.

FMQ: TD = unspecified DD**>ASD***>comorbidity = ID = motor dysfunction.

^a^ Comorbidity includes: ASD + ID (*N* = 98), ASD + ADHD (*N* = 72), ID + ADHD (*N* = 25), ASD + ID + ADHD (*N* = 17); PDMS-2, Peabody Developmental Motor Scales-Second Edition; VMI, visual motor integration; FMQ, fine motor quotient.

*** *p *< 0.001.

**Figure 1:**
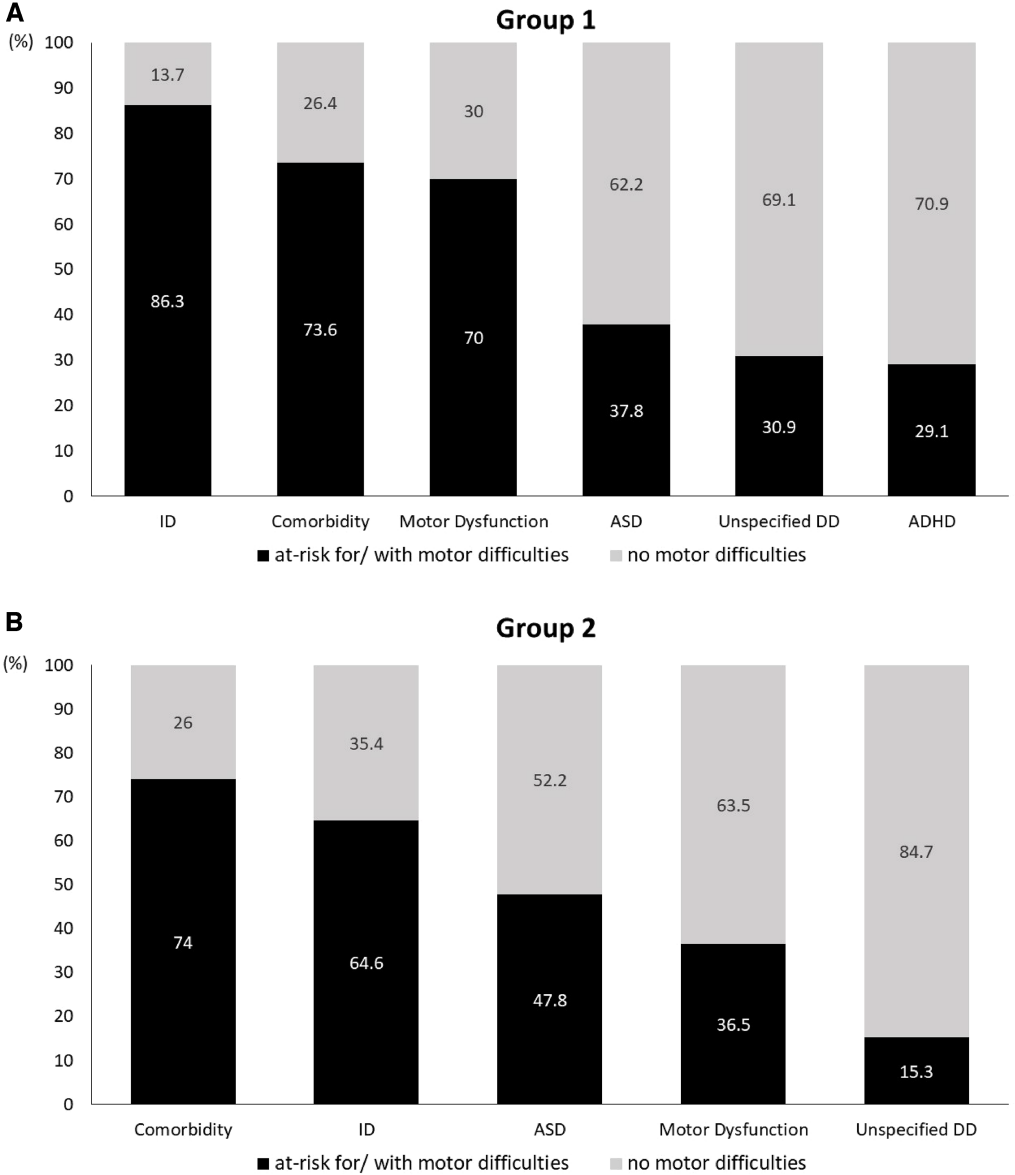
The distribution of the at risk for/with motor difficulties for different disability diagnoses.

In Group 1, children with ASD scored significantly higher in placing coins, threading beads, and drawing trials on the MABC-2 than those of children with ID, comorbidity, and motor dysfunction. Using a cutoff score of 7, almost one-third (37.8%) of the children with ASD were at risk for/with motor difficulties. Children with ID showed significantly lower scores for the performance of all subtests and total scores on the MABC-2 than those of the children with ASD, ADHD, comorbidity, and unspecified DD. The majority (86.3%) of children with ID were at risk for/with motor difficulties. Children with ADHD scored significantly better than children without ADHD on the MABC-2. Only 68 children with ADHD were at risk for/with motor difficulties. In terms of children with comorbidity, 73.6% were at risk for/with motor difficulties. They score significantly lower scores in all subtests and total scores on the MABC-2 than those of the children with ASD, ADHD, and unspecified DD. Almost 70% of the children with motor dysfunction were at risk for/with motor difficulties. Figures 1A,B shows the distribution of children at risk for/with motor difficulties, categorized by different disability diagnoses.

On the PDMS-2, 47.8% of the children with ASD scored a quotient below 85 (scored 1 SD below the mean), which was described as developmentally delayed. Children with ASD scored significantly better than children with ID, motor dysfunction, and comorbidity on the visual motor integration subtest and fine motor quotient. In terms of the PDMS-2, 64.6% of the children with ID scored as having a developmental delay. They scored significantly lower in the visual motor integration subtest and fine motor quotient than the children without ID.

A correlation matrix was used to illustrate the associations between cognitive ability and fine motor performance in the two groups. Positive correlations existed between IQ and placing coins (*r* = 0.445, *p *< 0.001), threading beads (*r* = 0.458, *p *< 0.001), drawing trials (*r* = 0.421, *p *< 0.001), and manual dexterity (*r* = 0.535, *p *< 0.001) in Group 1. For Group 2, positive correlations existed between cognitive ability and grasping (*r* = 0.374, *p *< 0.001), visual motor integration (*r* = 0.621, *p *< 0.001), and fine motor quotient (*r* = 0.571, *p *< 0.001). Table 3 presents the associations between cognitive ability and fine motor performance among children by different diagnoses. For both groups, there were no significant correlations between fine motor performance and birth order or smartphone usage.

**Table 3:** Intercorrelations of fine motor performance and cognitive ability for children with different disability diagnoses*.*

	Group 1						
Tests	ALL (*N* = 1,424)	ASD (*N* = 275)	ID (*N* = 124)	ADHD (*N* = 234)	Comorbidity (*N* = 212)	Motor dysfunction (*N* = 109)	Unspecified DD (*N* = 418)
MABC-2	WPPSI-IV	WPPSI-IV	WPPSI-IV	WPPSI-IV	WPPSI-IV	WPPSI-IV	WPPSI-IV
Placing coins	0.445***	0.257***	0.355**	0.269***	0.397***	0.378***	0.359***
Threading beads	0.458***	0.307***	0.342**	0.281***	0.460***	0.397***	0.308***
Drawing trail	0.421***	0.224***	0.337**	0.284***	0.421***	0.208*	0.286***
Manual dexterity	0.535***	0.359***	0.422***	0.355***	0.549***	0.423***	0.413***
	Group 2						
	ALL (*N* = 473)	ASD (*N* = 88)	ID (*N* = 99)	Comorbidity (*N* = 73)	Motor dysfunction (*N* = 19)	Unspecified DD (*N* = 172)	
PDMS-2	Bayley-III	Bayley-III	Bayley-III	Bayley-III	Bayley-III	Bayley-III	
Grasping	0.374***	0.138	0.485***	0.293*	0.197	0.165	
VMI	0.621***	0.302**	0.346**	0.437***	0.432**	0.346***	
FMQ	0.571***	0.320**	0.486***	0.480***	0.402**	0.264**	

PDMS-2, Peabody Developmental Motor Scales-Second Edition; VMI, visual motor integration; FMQ, fine motor quotient.

* *p* < .05.

** *p* < .01.

*** *p* < .001.

## 4 Discussion

This study adds to what is known about the differences in fine motor performance among young children with different disability diagnoses. Our findings indicate that many young children with ID, comorbidity, and motor dysfunction demonstrate significant difficulty in performing manual dexterity and visual motor integration and have significantly lower scores in these areas than children with ASD, ADHD, and unspecified DD. Additionally, fine motor performance was significantly correlated with cognitive ability, indicating that young children showed better fine motor performance when they demonstrated better cognitive ability.

Consistent with the findings of previous studies (8–13), the current study demonstrated that children with ASD, ID, and ADHD showed poorer fine motor performance than that of children with TD. As expected, children with comorbidity and motor dysfunction had significantly lower scores in manual dexterity than those of children with TD. These results support that children with comorbidity and motor dysfunction had difficulty adapting their movement to perform planned motion due to prominent behavioral features (15, 33). From the results, we observed that no difference between children with TD and unspecified DD is inconsistent with a previous study (4, 5). One reason might reflect the differences in the sample characteristics. In our research, the unspecified DD group contained mainly children with speech and language delays. Previous research revealed that the fine motor performance of children with speech and language delays did not differ from the normative sample (34). This reinforces the importance and benefit of considering fine motor performance comparisons between children with different developmental concerns and TD.

Although limited research provided evidence on children with comorbidity, our results are consistent with previous studies in which children with ASD co-occurring ID were significantly worse at manual dexterity than children with only ASD (22, 35). Indeed, children with comorbidity commonly demonstrate substantial heterogeneity and more impediments to functional capacity. In line with a previous study, our findings show that children with ASD and co-occurring ADHD or ID score significantly lower than children with only ASD diagnosis (22). One reason might be that more severe difficulties with attentional deficits, visual perception, and executive functioning in children with ASD and ADHD or ID were evident compared with children with ASD (36–38). Rather surprisingly, the findings from this study did not correspond with previous research (13), where a significantly lower score was found in children with ASD than children with ADHD or ID (14). It appears that there were contrasting results in our study, and a possible explanation for this might be the heterogeneous samples in the previous studies (13, 14). Though those ADHD or ID groups were the primary diagnosis of all individuals in the recruited group, some children with co-occurring developmental coordination disorder or motor speech impairments have not been excluded for analysis.

Notably, weak to moderate correlations were observed between fine motor performance and cognitive ability in children with different disability diagnoses. These results align with previous studies, highlighting that an impediment in cognitive abilities was related to poor fine motor performance among children with developmental disabilities (6, 21–24, 39). The results in this study extend the current knowledge of determining links between fine motor performance and cognitive ability in children with ASD co-occurring ID or ADHD. Furthermore, Ramos-Sánchez et al. (22) and Surgent et al. (40) indicated that cognitive ability rather than diagnostic groups may be best predictive of fine motor performance. Thus, the results support that cognitive ability contributes to performing fine motor skills for children with developmental disabilities (18–20). However, our findings did not correspond to the findings of Asonitou et al. (25), who reported that cognitive abilities (planning and attention) were not associated with manual dexterity for children with developmental coordination disorder. Only working memory was related to manual dexterity. Thus, it is plausible to assume that there is a relationship between cognitive ability and visual motor integration, which requires planning and execution for children with motor dysfunction. This finding might be attributable to the heterogeneity characterized by varying degrees of motor dysfunction. Some children had gross or fine motor deficits, whereas some had combined gross and fine motor deficits in this study. Therefore, this finding indicated that visual motor integration is specifically impaired in children with motor dysfunction and further highlights the importance of adequately controlling for sample characteristics while taking into account cognitive ability (such as specific executive functioning).

This study has certain limitations. First, children were recruited from southern cities in Taiwan. Therefore, the findings of this study may not be generalized to geographical differences in socioeconomic status in other areas of Taiwan. Second, reliability and normative data of motor assessments for children with ASD and comorbidity are lacking. Third, applying a standardized assessment procedure would be questionable in examining motor skills in children with severe and profound intellectual disability. Lastly, we conducted a retrospective cohort study; though the sample size was large, the number of samples for each disability type was uneven. Despite the inherent limitations of this study, the findings provide valuable information on the fine motor performance and cognitive ability of young children with different disability diagnoses and crucial associations between fine motor performance and cognitive ability. As a result, this study extends the research findings on young children with developmental disabilities under the age of 6 years.

## 5 Conclusion

Following the results of our study, we can conclude that the fine motor performance of some children with developmental disabilities differed from that of TD children. Furthermore, young children with ID, comorbidity, and motor dysfunction demonstrated significant difficulty in performing manual dexterity and visual motor integration tasks. They scored significantly lower in these areas compared to children with ASD, ADHD, and unspecified DD. The study provides a comprehensive understanding of the fine motor performance of young children with different disability diagnoses. Furthermore, it was observed that children with better cognitive abilities exhibited better fine motor performance. These findings reveal that early intervention focused on fine motor and cognitive skills is crucial for children with developmental disabilities. Pediatric clinicians should consider cognitive ability when evaluating and intervening in fine motor performance. They should be aware of the role that cognitive ability in early childhood plays in predicting persistent fine motor impairment. Further research using targeted cognitive ability could help determine the mechanism and nature of fine motor difficulties observed in children with different disability diagnoses.

## Data Availability

The original contributions presented in the study are included in the article/Supplementary Material; further inquiries can be directed to the corresponding authors.
